# External ears for non-invasive and stable monitoring of volatile organic compounds in human blood

**DOI:** 10.1038/s41598-021-90146-1

**Published:** 2021-06-10

**Authors:** Koji Toma, Shota Suzuki, Takahiro Arakawa, Yasuhiko Iwasaki, Kohji Mitsubayashi

**Affiliations:** 1 Department of Biomedical Devices and Instrumentation, Institute of Biomaterials and Bioengineering, Tokyo Medical and Dental University, 2-3-10 Kanda-Surugadai, Chiyoda-ku, Tokyo, 101-0062 Japan; 2 Graduate School of Medical and Dental Sciences, Tokyo Medical and Dental University, 1-5-45 Yushima, Bunkyo-ku, Tokyo, 113-8510 Japan; 3 Faculty of Chemistry, Materials and Bioengineering, Kansai University, Osaka, Tokyo 113-8668 Japan

**Keywords:** Biomedical engineering, Health care

## Abstract

Volatile organic compounds (VOCs) released through skin (transcutaneous gas) has been increasing in importance for the continuous and real-time assessment of diseases or metabolisms. For stable monitoring of transcutaneous gas, finding a body part with little interference on the measurement is essential. In this study, we have investigated the possibility of external ears for stable and real-time measurement of ethanol vapour by developing a monitoring system that consisted with an over-ear gas collection cell and a biochemical gas sensor (bio-sniffer). The high sensitivity with the broad dynamic range (26 ppb–554 ppm), the high selectivity to ethanol, and the capability of the continuous measurement of the monitoring system uncovered three important characteristics of external ear-derived ethanol with alcohol intake for the first time: there is little interference from sweat glands to a sensor signal at the external ear; similar temporal change in ethanol concentration to that of breath with delayed peak time (avg. 13 min); relatively high concentration of ethanol relative to other parts of a body (external ear-derived ethanol:breath ethanol = 1:590). These features indicated the suitability of external ears for non-invasive monitoring of blood VOCs.

## Introduction

Exhaled breath and transcutaneous gas contain volatile organic compounds (VOCs) from blood^1^. Some of these blood VOCs are resultant product of diseases or metabolisms, which has been attracting great attention to utilize these VOCs for non-invasive and simple disease screening and metabolism assessment^2,3^. For example, acetone is a lipid metabolite and expected for its application in lipid metabolism assessment and diabetes screening^4^.

Transcutaneous gas is more suitable to real-time and continuous assessment than breath due to two reasons: one is that the transcutaneous gas is released unconsciously and continuously; the other is that there is much less limitations on time and places for the collection of gas sample than breath that requires a mask or complicated procedures, such as collecting end-tidal air^5,6^.

There are highly sensitive analytical methods for VOCs in exhaled breath and transcutaneous gas, such as gas chromatography mass spectrometry (GC–MS)^7^, proton-transfer-reaction mass spectrometry (PTR-MS)^8^ that is often coupled with time of flight mass analyser (PTR–TOF–MS)^9^, and selected-ion flow-tube mass spectrometry (SIFT-MS)^10^. Although their capability of identifying VOC composition is powerful for comprehensive analysis of the gas, the size and complexity of the system are drawbacks for monitoring transcutaneous VOC.

As simpler methodologies for blood VOCs, sensors are broadly and intensively studied from academia to industry. Lansdrop et al. developed a wristband wearable alcohol biosensor which used alcohol oxidase and Prussian Blue to measure transcutaneous blood ethanol electrochemically^11^. Kim et al. reported a wearable tattoo-based alcohol monitoring system which demonstrated measurement of sweat ethanol on a skin using amperometry with alcohol oxidase and Prussian Blue^12^. Yamada et al. presented a semiconductor-based portable sensor for transcutaneous blood acetone, which utilized zeolite to concentrate acetone before measurement^13,14^.

One of the biggest challenges for measurement of transcutaneous blood VOCs is its low concentration relative to breath. Generally, blood/breath alcohol concentration ratio of 2100/1 is used for legal purposes. Transcutaneous ethanol collected by Nalophan bags showed the emission rate of 48 ppt/(cm^2^∙min) without alcohol intake, whereas the concentration in breath was 37–207 ppb^15,16^. Both concentrations increase when drinking alcohol together with blood alcohol concentration. Our previous study revealed that the ethanol concentrations in transcutaneous gas from a whole hand and exhaled breath were 46 ppb/cm^2^ and 47 ppm, respectively, with alcohol intake^17^. These data indicate that transcutaneous/breath ethanol concentration ratio is approximately 1/1000.

Another difficulty to measure transcutaneous blood VOCs is interference by sweat and different release dynamics of the gas depending on a body part because each body part has different density of sweat glands and epidermis layers of the skin; therefore, it is important to choose a proper body region to acquire relatively high concentration of a target VOC with little interference by sweat for monitoring transcutaneous blood VOCs.

The external ear region has a skin with lower density of sweat glands (140 spots/cm^2^) compared to other regions of a body, such as a palm (620 spots/cm^2^), forearm (225 spots/cm^2^) and cheek (360 spots/cm^2^)^18,19^. In addition, Lobitz et al. reported that an external ear canal has no eccrine sweat gland^20^. Also, gas from the external ear is not only from an outer part of the external ear but also from the external ear canal^21^. It possibly causes the transcutaneous VOCs in the middle ear to be released through the tympanic membrane to the external ear canal^22–24^ and the concentration of VOCs at the external ear to be high. In addition, the external ear has a relatively large space to incorporate devices to measure.

Based on the reasons above, we hypothesized that an external ear is a promising candidate for stable monitoring transcutaneous blood VOCs. To examine this hypothesis, this study began with the development of a monitoring system for external ear-derived ethanol, then the system was applied for real-time and continuous measurement of ethanol at the external ear with alcohol intake.

## Results and discussion

### Construction of the monitoring system

**Figure 1:**
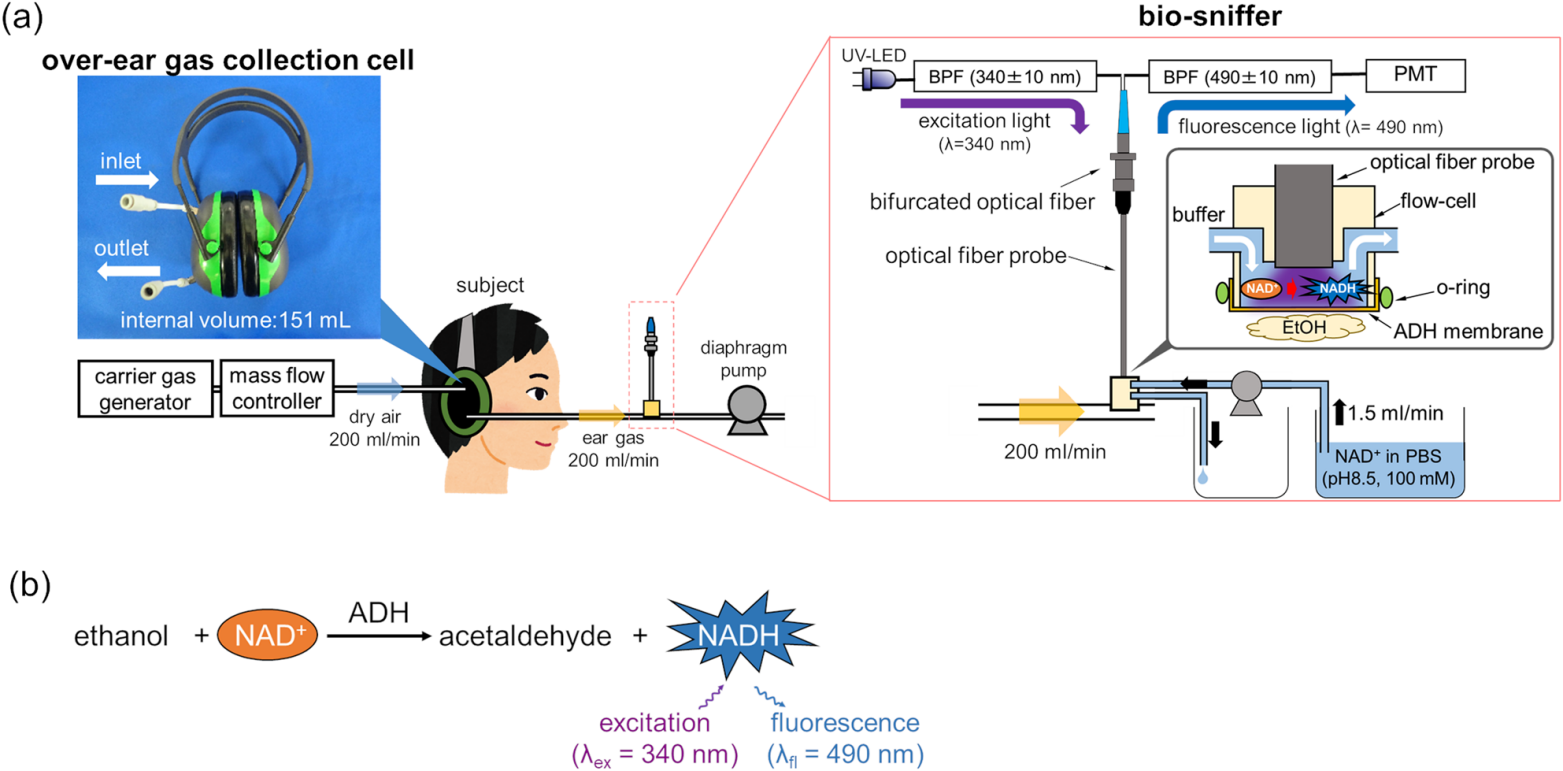
(**a**) A schematic illustration of the monitoring system for external ear-derived ethanol. The system was composed of the over-ear gas collection cell and bio-sniffer. (**b**) A principle of ethanol measurement using ADH and NADH.

The monitoring system was developed by combining an over-ear gas collection cell and a gas-phase biosensor (bio-sniffer) for ethanol (Fig. 1a). The bio-sniffer measures ethanol vapor based on the principle shown in Fig. 1b. Alcohol dehydrogenase (ADH) catalyses oxidation of ethanol to produce acetaldehyde. Simultaneously, a coenzyme, oxidized form of β-nicotinamide adenine dinucleotide (NAD^+^), accepts the electron to become the reduced form (NADH) that exhibits autofluorescence (λ_ex_ = 340 nm, λ_fl_ = 490 nm); therefore, ethanol can be measured by detecting the increase in the autofluorescence from NADH. An enzyme-immobilized membrane (ADH membrane) attached on the flow-cell worked as a gas–liquid diaphragm. When the ethanol vapor reaches the ADH membrane, the catalysed redox reaction occurs at the membrane together with NAD^+^ in the buffer solution running above the membrane, which produces NADH in the buffer solution. Resultant NADH was excited by the UV light from the optical fiber probe, then the emitted fluorescence was collected by the same fiber probe to be detected. In the monitoring of external ear-derived gas, the sample gas from a subject was collected by the over-ear gas collection cell, then simultaneously transported to the bio-sniffer for the real-time measurement.

### Characterization of ethanol bio-sniffer and over-ear gas collection cell

**Figure 2:**
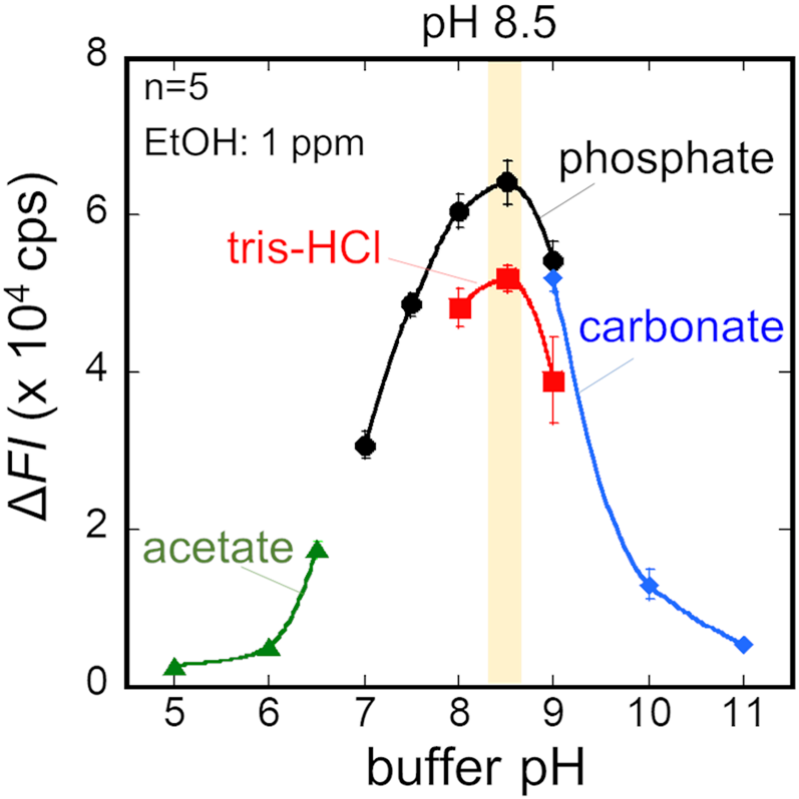
Influence of the buffer pH on the sensor output (Δ*FI*).

In order to investigate the influence of buffer solution pH on the activity of ADH, four different buffer solutions, including acetate buffer (AB), phosphate buffer (PB), tris–HCl and carbonate-bicarbonate (CB) solutions, were pumped to the flow-cell in the ethanol bio-sniffer at the flow rate of 1.5 mL/min while measuring 1000 ppb standard gaseous ethanol. The fluorescence intensity increased when the gaseous ethanol was loaded to the ethanol bio-sniffer, indicating that NADH was produced by ADH-catalyzed redox reaction of ethanol as described in the Fig. 1b. Then, the fluorescence intensity reached plateau under the balance between removal of produced NADH and supply of NAD^+^, which was controlled by the flow rate of a buffer solution. When switching the gaseous ethanol to the carrier gas, the fluorescence intensity decreased because of the termination of the ADH-catalyzed reaction and removal of NADH. Here, the sensor output (Δ*FI*) was defined as an increment of the fluorescence intensity from the baseline. The sensor output for each buffer solution is presented in Fig. 2. The output peaks with pH 8.5 PB solution. Therefore, pH 8.5 PB solution has used for subsequent experiments.

**Figure 3:**
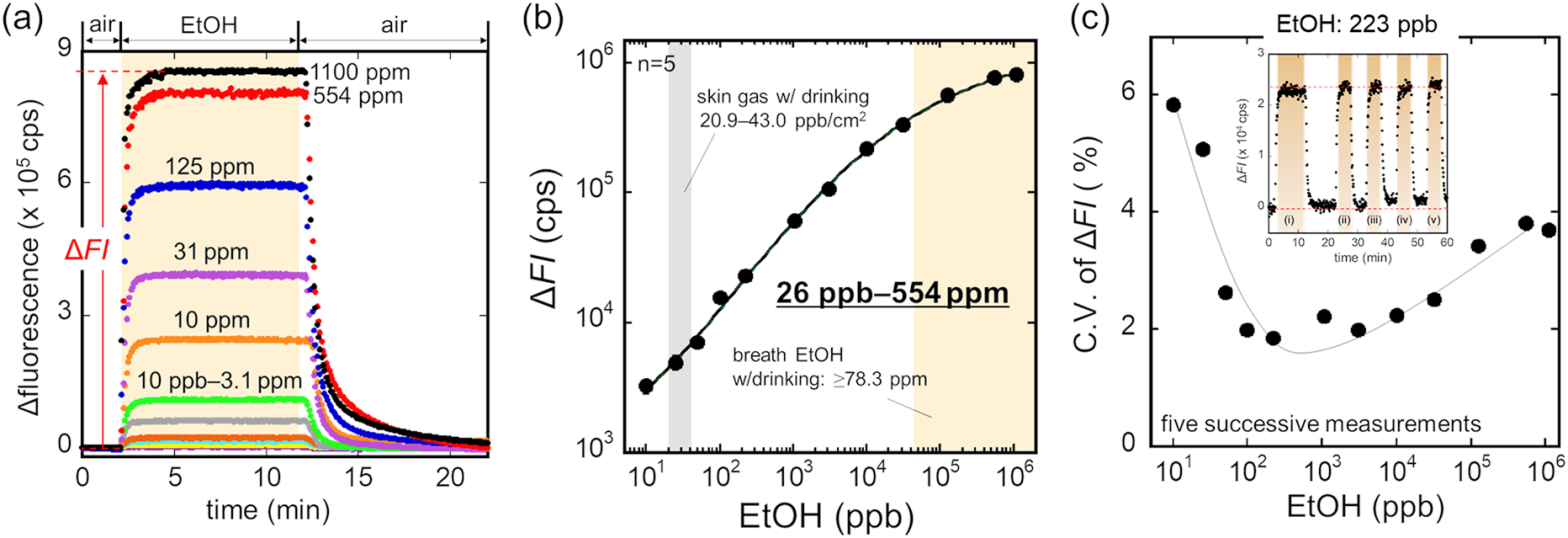
(**a**) Sensor responses to standard gaseous ethanol with various concentrations. (**b**) Calibration curve of the bio-sniffer to gaseous ethanol. (**c**) Sensor output variations in 5 repeated measurement of gaseous ethanol with different concentrations. The inset shows the sensor responses to 223 ppb ethanol.

The sensitivity of the ethanol bio-sniffer and the reproducibility of the sensor output for various ethanol concentrations were investigated. Figure 3a shows the sensor responses to the gaseous ethanol with the concentrations from 10 ppb to 1100 ppm. The sensor output was dependent on the concentration of gaseous ethanol. A calibration curve in Fig. 3b shows the dynamic range of the ethanol bio-sniffer is 26 ppb–554 ppm, which was determined from the intersection where ten times a standard deviation of the baseline obtained from the carrier gas equals the sensor output. The reproducibility of the sensor output during five repeated measurement of gaseous ethanol, whose concentrations correspond to those of the calibration curve, is shown in Fig. 3c.

The reproducibility was assessed for each concentration by calculating the average coefficient of variation (C.V.) of the sensor output from five trials. It shows the highest C.V. of about 4–6% at the lowest and highest concentrations, while the lowest C.V. of about 2% appeared in the middle concentration range.

**Figure 4:**
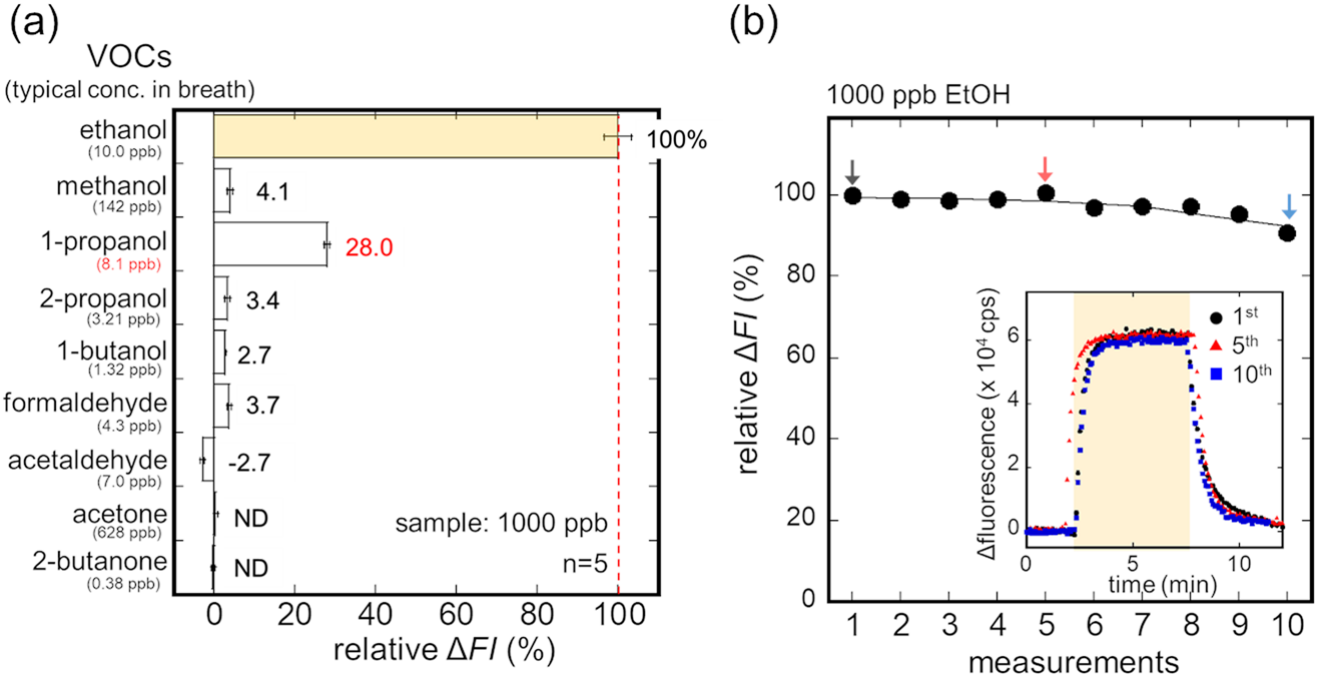
(**a**) Relative sensor output to representative VOCs in breath. (**b**) Relative sensor output during 10 repeated measurement of 1000 ppb gaseous ethanol.

The selectivity of the ethanol bio-sniffer was assessed with representative VOCs in breath, including ethanol, methanol, 1-propanol, 2-propanol, 1-butanol, formaldehyde, acetaldehyde, acetone, and 2-butanone. The concentrations of all the VOCs were fixed to be 1000 ppb. Relative sensor outputs to that from ethanol are shown in Fig. 4a. Except for 1-propanol, the relative outputs were as small as or smaller than a few per cent. Considering that breath ethanol concentration increases to about 100 ppm when drinking alcohol, even the output from 1-propanol can be ignored because a typical concentration of 1-propanol in breath is 8.1 ppb^25^. These results supported the high selectivity of the bio-sniffer, relying on ADH specificity, to gaseous ethanol, and neglectable interference from other compounds in the gas mixture would be expected. However, there are still possibilities of interreferences which did not appear in the relative output to each compound one by one, for example unexpected inhibition or enhancement of the signal to ethanol, and they will be investigated in future study using mixed gas samples.

Presence of interferential VOCs in gas released from the over-ear gas collection cell was investigated. The gas collection cell was sealed by a polymethyl methacrylate plate. A sensor response to the gas from the collection cell exhibited no change in the fluorescence intensity when the carrier gas was exchanged to that from the gas collection cell. This result proved that no interferential VOCs are present in the gas from the over-ear gas collection cell.

The repeatability and stability of the sensor signal were also assessed by ten successive measurement of 1000 ppb gaseous ethanol. The sensor output was not significantly degraded during 10 repeated measurements taking about 150 min (see Fig. 4b). The inset in Fig. 4b also clearly shows that the sensor responses are nicely overlapped. These results indicate that the bio-sniffer can be used in continuous measurement of gaseous ethanol.

### Monitoring of external ear-derived ethanol after drinking alcohol

**Figure 5:**
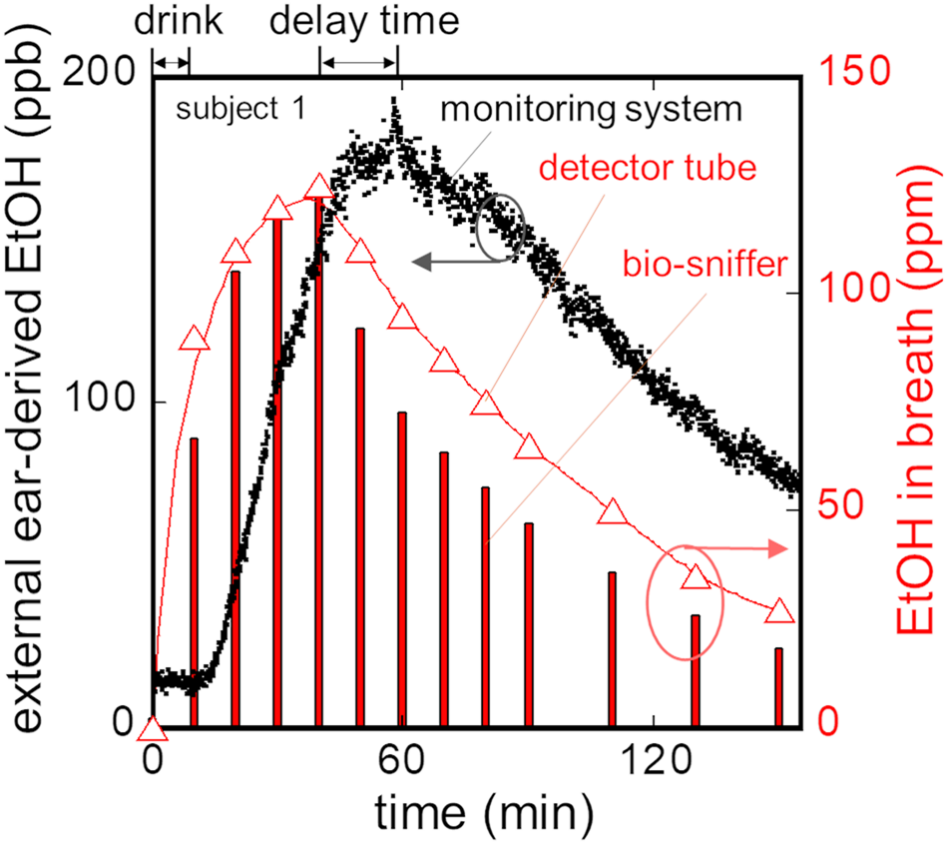
Monitoring of ethanol concentration after alcohol intake in the ear gas by the developed system for the subject 1. The ethanol concentration in the ear gas (●); in the breath measured by (Δ) detector tubes and (bars) the ethanol bio-sniffer.

The developed monitoring system was applied to real-time measurement of external ear-derived ethanol. Figure 5 shows a temporal change of ethanol in the external ear gas along with that in breath measured by detector tubes (bars) and the ethanol bio-sniffer (triangles). The ethanol concentration from the external ear started to rise 7 min after alcohol intake and reached the peak of 183 ppb 52 min later, then the ethanol concentration gradually decreased by alcohol metabolism. This temporal change has a similarity to that of breath ethanol concentration. Here, the validity of the breath ethanol concentrations was supported by close values and the same temporal changes obtained by two different methods. According to other reports proving that ethanol concentrations in blood and breath have a correlation, the concentration of the external ear’s ethanol is most likely correlated to that in blood^26–28^.

Another important finding in this result was much lower noise in the signal than that from a palm^17^. The noise level was evaluated by a moving average and standard deviation for the change in the external ear-derived ethanol concentration over time, with 5 min as an interval (see Supplementary Fig. S1 online). Using these average and standard deviation, C.V. of the moving average of the ethanol concentration was also calculated. It was found the average C.V. from 0 to 90 min was of 6.9%. The same analysis was conducted to the ethanol concentration from a palm presented in our previous study^17^, and it resulted in the C.V. of 22.7%. These C.V. indicate that measuring ethanol with the external ear reduced the noise by a third compared to the palm. The reason of this low noise is assumed to be that there are few eccrine sweat glands on an external ear canal while a palm has eccrine sweat glands most densely through a body, 620 spots/cm^2^ ^18,20^. A comparison of sweat rate between a palm and external ear for another subject without alcohol is shown in Supplementary Fig. S2 online. The sweat rate of a palm (171.4 g/(m^2^∙h)) was well agreed to the reports by Park et al. (109.15 g/(m^2^∙h))^29^ and Taylor et al. (197 g/(m^2^∙h))^19^. The sweat rate in the external ear canal (103.7 g/(m^2^∙h)) was relatively higher than those of a wrist (24.6 g/(m^2^∙h)) and earlobe (38.1 g/(m^2^∙h)). This is perhaps due to apocrine sweat glands distributed in the outer one-third of the external ear canal^30^. Another reason may be connection to deeper part in the ear, which allows sweat and moisture in the deeper region to be exhaled through the external ear canal. On the other hand, the sweat rate of a palm largely fluctuates with the C.V. of 14.9%, while that of an external ear canal was stable with the C.V. of 5.0%. Since the sweat rates of the palm and wrist were similar to our previous studies, where the stability of ethanol concentrations had been influenced by the sweat rates, it was suggested that the stable ethanol concentration in Fig. 5 was related to stable sweating in the external ear canal^17,31^. To further reduce the noise in the external ear-derived ethanol or reduce it in the palm-derived, the shapes of collection cells may be considered in future study.

The same experiment was carried out for two other subjects. Although there were individual variations, the same trends, such as similar temporal change of ethanol concentration in the ear gas to that of breath ethanol along with delayed peak time of the ear gas from breath, were observed. A summary of ethanol concentrations in the ear gas and breath is presented in Table 1. The average peak ethanol concentrations in the ear gas and breath were 148 ± 29 ppb and 87 ± 34 ppm, respectively, which leads to the concentration ratio of the ear gas to breath of 1:590. A statutory limit of breath alcohol concentration for driving in Japan is 78.3 ppm, and the breath concentration after intake of 0.6 g per kg body weight alcohol was 114.7 ppm according to a study of Lindberg et al.^27^; they support the validity of the concentration of breath ethanol determined in this study. Our previous study revealed that the ethanol concentration from a hand (palm: 46 ppb/cm^2^; wrist: 20.9 ppb/cm^2^) was about 1000-fold smaller than that in exhaled breath (47.1 ± 8.2 ppm)^17^. Therefore, the ethanol concentration in the ear gas is higher than the ethanol level from the hand emission. Please note that ethanol concentration of a hand was calculated by dividing the measured concentration by the area of a gas collection cell attached on a hand, while the ethanol concentration of an external ear is the total collected by the over-ear gas collection cell.

**Table 1:** Comparisons of ethanol concentrations in external ear gas and breath.

Subject	Ethanol conc. (ppb)		Ethanol conc. ratio (ear gas/breath)	Delay time (min)
	Ear gas	Breath		
A	186	122,000	1/650	17
B	116	98,000	1/840	10
C	142	40,000	1/280	12
Avg.	148 ± 29	87,000 ± 34,422	1/590	13 ± 3

The average peak time of the external ear-derived ethanol concentration was 13 min-delayed from that in the breath. It was a similar result to other transcutaneous gases^17,32^. The delay was probably due to a thickness differences of layers which ethanol passes through. The thickness of a blood-air barrier of an alveolus was 2.2 μm while the thickness of an external ear’s epidermal layers is 69.3 μm^33,34^; therefore, diffusion and transmission of VOCs from a blood vessel is slower in the ear gas.

In this study, the measurement was made in one ear which we randomly selected because it was supposed that both external ear canals were symmetric and have no differences in ethanol concentrations. However, it is also possible to measure ethanol concentrations in parallel not only in both ears but also different body parts by adding more bio-sniffers to the monitoring system.

Conventionally, ethanol, particularly in exhaled breath, is measured by colorimetry, infrared (IR) spectroscopy, and fuel cell technology. These methodologies have been already implemented in products and used in practice^35^. However, there is an interference problem from other alcohols. For example, Laakso et al. studied the interference from solvents on ethanol analysis by an IR spectroscopy-based evidential breath analyser. Their study revealed that ketones, diethyl ether and ethyl acetate did not interfere with breath ethanol measurement, but 1-propanol and 2-propanol had a significant influence on the ethanol measurement^36^. Our sensor, on the other hand, relies on an enzyme that has high specificity to a substrate. Therefore, as presented in Fig. 4a, there is no significant interference from other alcohols. In addition, the capability of continuous measurement is suitable for the monitoring. These two unique properties of our monitoring system enabled us to explore the possibility of external ears for blood VOC monitoring.

In this study, ethanol was selected as a model target VOC, but principally this system is applicable to other VOCs by changing an enzyme in the bio-sniffer. Therefore, in future, the monitoring system will be applied to metabolisms assessment and disease screening that require detailed and real-time temporal information.

## Conclusion

We have examined the possibility of the external ear for stable monitoring of transcutaneous VOC by developing the monitoring system. The monitoring system comprises of the over-ear gas collection cell and the ethanol bio-sniffer and allowed for real-time and continuous measurement of temporal change in ethanol concentration of the ear gas after alcohol intake. This monitoring uncovered three important characteristics of the ear-derived ethanol for the first time: little interference from sweat to the sensor signal; higher ethanol concentration than that from a hand; similar temporal change to the breath with delayed peak time. These findings suggest the suitability of the external ear for blood ethanol monitoring. The monitoring system is potentially applicable to other VOCs b changing an enzyme. Using this versatility, we will further investigate external ear-derived VOCs for non-invasive and real-time assessment of metabolisms and disease screening.

## Methods

### Materials

ADH (activity of 128 unit/mg), and oxidized form of β-nicotinamide adenine dinucleotide (NAD^+^) were purchased from Oriental Yeast (Japan). A hydrophilic polytetrafluoroethylene (H-PTFE) membrane (thickness of 80 μm, porosity of 80%, pore size of 0.2 μm) used for an enzyme membrane was from Millipore (USA). A polymer entrapping ADH in the membrane, poly[2-methacryloyloxyethyl phosphorylcholine (MPC)-co-2-ethylhexyl methacrylate (EHMA)] (PMEH), was synthesized in house by free radical-polymerization method^37^.

All the chemicals to prepare the following three different buffer solutions were from Fujifilm Wako Pure Chemical (Japan). PB solution (100 mM, pH 8.0) was prepared by adding potassium dihydrogen phosphate solution (100 mM in ultrapure water) to disodium hydrogen phosphate solution (100 mM in ultrapure water) to buffer the solution pH to 8.0. AB solution (100 mM) was prepared by adding 100 mM sodium acetate solution to 100 mM acetic acid solution to buffer the pH to 5.0–6.5. Tris–HCl solution (100 mM) was prepared to add HCl solution to 100 mM trimethylolaminomethane solution to buffer the pH to 8.0–9.0. 100 mM CB solution (100 mM) was prepared to add 100 mM sodium carbonate solution to sodium hydrogen carbonate solution to buffer pH to 9.0–11.0.

### Construction of ethanol bio-sniffer

The bio-sniffer was composed of excitation and detection units which were connected to a bifurcated optical fibre (PVSMA2-2 STU600-STUH190S, one fibre with the core diameter of 600 μm for fluorescence surrounded by 35 fibres with the core diameter of 190 μm for ultraviolet light, Mitsubishi Cable Industries, Japan). The excitation unit has a UV light emitting diode (UV-LED, centre wavelength = 340 nm, 340-FL-01-G01, DOWA, Japan) and a bandpass filter (BPF, λ = 340 ± 10 nm, LX0340, Asahi Spectra, Japan) to make the peak wavelength of the excitation light 340 nm. The detection unit has a photomultiplier tube (PMT, H7421-40, Hamamatsu Photonics, Japan) and another BPF (λ = 490 ± 10 nm, MX0490, Asahi Spectra, Japan) to extract fluorescence light from NADH. A flow-cell made of polymethyl methacrylate is attached to an optical fibre probe end (F1000-ANGLE90, Ocean Optics, USA), that is connected to the bifurcated optical fibre. ADH membrane was attached and fixed at the end of the flow-cell by an o-ring to work as a gas–liquid diaphragm.

The ADH membrane was prepared as follows: ADH was dissolved in PB (pH 8.0, 80 mM) to prepare 4.7% (w/v) ADH solution; a 15% (w/w) PMEH solution was prepared using ethanol; a mixed solution (60 units/cm^2^) of the ADH and PMEH solutions at the same volume ratio was spread over the 2 × 2 cm^2^ H-PTFE membrane; the coated PTFE membrane was dried for 3 h followed by rinsing with PB.

### Fabrication of over-ear gas collection cell for external ear’s transcutaneous ethanol

An over-ear gas collection cell was fabricated by modifying a commercial earmuff (X1A, 3 M) which has cups made of acrylonitrile-styrene-acrylate resin and urethane cushions covered by polyvinyl chloride sheets. Two connectors with the inner diameter of 4 mm were attached to the holes made on the earmuff cups. These holes were used as an inlet for a filtered air (carrier gas) and an outlet for sample gas containing ear gas, respectively (Fig. 1a). The internal volume of the cup is 151 mL.

### Monitoring of external ear-derived ethanol after alcohol intake

Real-time and continuous measurement of external ear-derived ethanol was conducted for three healthy male subjects with alcohol intake. This experiment was approved by the ethics committee of Faculty of Medicine, Tokyo Medical and Dental University (approval number: M2018-160) and performed in accordance with the guidelines and regulations after informed consent had been obtained from all subjects. The subjects did not take any alcohol and drugs for 72 h before the experiment.

The carrier gas from a compressor flew into the collection cell attached on a subject’s ear at the flow rate of 200 mL/min and was pumped out with the same flow rate by a diaphragm pump located behind the bio-sniffer in order to make the internal pressure of the cell unchanged. The side of the ear being measured was randomly selected.

Firstly, base ethanol concentration in the external ear gas was measured for 10 min without drinking alcohol. Then, a subject drank alcohol at the concentration of 0.4 g per kg body weight within 5 min, and the measurement continued for another 140 min.

For comparisons, ethanol concentrations in breath were also simultaneously but intermittently measured by detector tubes (GASTEC, accuracy: C.V. of 10% for 100 to 500 ppm, Japan) and the ethanol bio-sniffer. The end-expiratory sampling method was employed to collect breath samples. In the method, a subject inhaled deeply though the nose and paused for 10 s; then, the subject exhaled through the mouth gently for 3 s to exclude the dead space followed by breathing into a gas sampling bag with the volume of 3 L. The inside of the gas sampling bag was fluorine-coated to prevent adsorption of breath components.

### Sweat rate measurement

This experiment was approved by the ethics committee of Faculty of Medicine, Tokyo Medical and Dental University (approval number: M2018-160) and performed in accordance with the guidelines and regulations after informed consent had been obtained from a subject. Sweat rates of four different parts (palm, wrist, external ear canal, and earlobe) were measured with a ventilated capsule-type perspiration meter (SKN-2000, Nishizawa Electric Meters Manufacturing, Japan). A capsule of the perspiration meter was attached on a skin surface, and the sweat rate was measured for 5 min at room temperature. A male subject who had fasted for 6 h and had not consumed alcohol was at rest during the measurement.

## Supplementary Information

Supplementary Information.
