# Supplementary material for: External ears for non-invasive and stable monitoring of volatile organic compounds in human blood
Source: Sci Rep. 2021 Jun 10;11:10415. doi: 10.1038/s41598-021-90146-1 (PMC8192764; doi:10.1038/s41598-021-90146-1)
Supplement: Supplementary file 1 — Supplementary Information. [file 41598_2021_90146_MOESM1_ESM.pdf]

Supplementary Information

External ears for non-invasive and stable monitoring of volatile organic compounds in human blood

Koji Toma[a], Shota Suzuki[b], Takahiro Arakawa[a], Yasuhiko Iwasaki[c], Kohji Mitsubayashi[a,b,*]

[a] *Department of Biomedical Devices and Instrumentation, Institute of Biomaterials and Bioengineering, Tokyo Medical and Dental University, 2-3-10 Kanda-Surugadai, Chiyoda-ku, Tokyo 101-0062, Japan*

[b] *Graduate School of Medical and Dental Sciences, Tokyo Medical and Dental University, 1-5-45 Yushima, Bunkyo-ku, Tokyo 113-8510, Japan2*

[c] *Faculty of Chemistry, Materials and Bioengineering, Kansai University, Osaka, Japan, Tokyo 113-8668, Japan*

** Corresponding author. Tel.: +81 3 5280 8091, Fax: +81 3 5280 8094*

E-mail: m.bdi@tmd.ac.jp

Supplementary Information

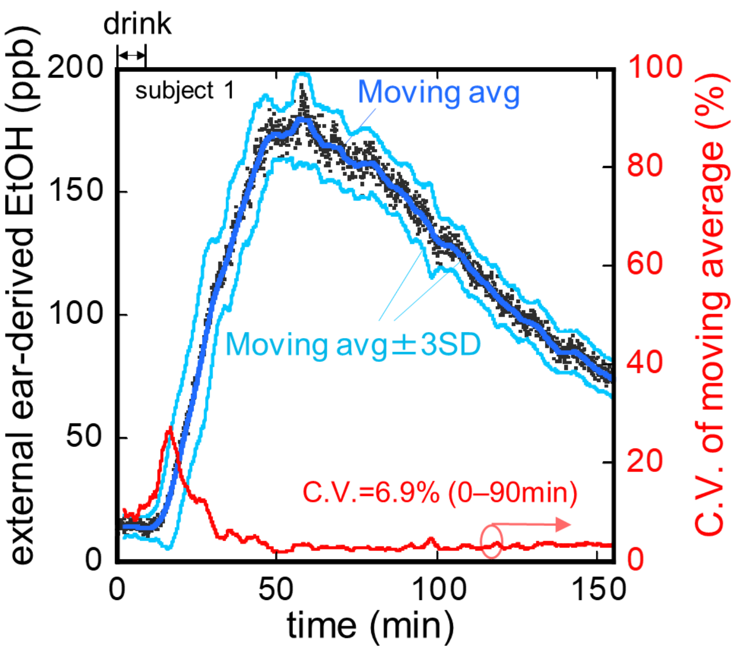

Fig. S1 Moving average of external ear-derived EtOH concentration (from Fig. 5) and their standard deviation (SD) and coefficient of variation (C.V.). The C.V. was calculated by the moving average and SD. The interval for the moving average and SD was 5 min.

Supplementary Information

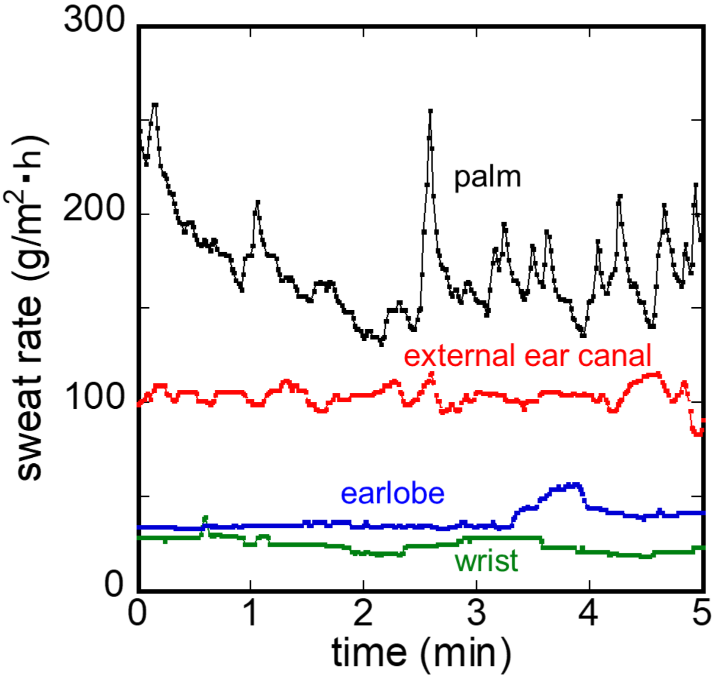

Fig. S2 Sweat rate for various parts of a body (palm, wrist, external ear canal, and earlobe) without consuming alcohol.
